# Attitudes of Health Professional Educators Toward the Use of Social Media as a Teaching Tool: Global Cross-Sectional Study

**DOI:** 10.2196/mededu.6429

**Published:** 2017-08-04

**Authors:** Karan D'Souza, Lucy Henningham, Runyu Zou, Jessica Huang, Elizabeth O'Sullivan, Jason Last, Kendall Ho

**Affiliations:** ^1^ Faculty of Medicine University of British Columbia Kelowna, BC Canada; ^2^ School of Health Sciences University of Melbourne Melbourne Australia; ^3^ School of Public Health Fudan University Shanghai China; ^4^ School of Dentistry University of Birmingham Birmingham United Kingdom; ^5^ School of Medicine University College of Dublin Dublin 4 Ireland; ^6^ Faculty of Medicine University of British Columbia Vancouver, BC Canada

**Keywords:** health education, health survey, teaching, health knowledge, attitudes, practice, interdisciplinary studies, social media, faculty development

## Abstract

**Background:**

The use of social media in health education has witnessed a revolution within the past decade. Students have already adopted social media informally to share information and supplement their lecture-based learning. Although studies show comparable efficacy and improved engagement when social media is used as a teaching tool, broad-based adoption has been slow and the data on barriers to uptake have not been well documented.

**Objective:**

The objective of this study was to assess attitudes of health educators toward social media use in education, examine differences between faculty members who do and do not use social media in teaching practice, and determine contributing factors for an increase in the uptake of social media.

**Methods:**

A cross-sectional Web-based survey was disseminated to the faculty of health professional education departments at 8 global institutions. Respondents were categorized based on the frequency of social media use in teaching as “users” and “nonusers.” Users sometimes, often, or always used social media, whereas nonusers never or rarely used social media.

**Results:**

A total of 270 health educators (52.9%, n=143 users and 47.0%, n=127 nonusers) were included in the survey. Users and nonusers demonstrated significant differences on perceived barriers and potential benefits to the use of social media. Users were more motivated by learner satisfaction and deterred by lack of technology compatibility, whereas nonusers reported the need for departmental and skill development support. Both shared concerns of professionalism and lack of evidence showing enhanced learning.

**Conclusions:**

The majority of educators are open-minded to incorporating social media into their teaching practice. However, both users and nonusers have unique perceived challenges and needs, and engaging them to adapt social media into their educational practice will require previously unreported approaches. Identification of these differences and areas of overlap presents opportunities to determine a strategy to increase adoption.

## Introduction

Social media is an inexpensive, powerful, and influential way of using Internet-based tools to facilitate easy and broad communication and the sharing of information and opinions [1,2]. Given the requirement of strong communication skills to provide excellence in health care and the rapid growth of social media usage as a medium of communication, health providers need to understand and adapt to social media use as a potential method of interacting with patients to provide care that meets the public’s needs and expectations [1-3].

Currently, up to 70% of the general public seeks health care information and advice from Internet sources, and they continue to use Web-based resources to strengthen their capacity to communicate about their health needs [4]. Whereas penetration in North America, Europe, and Australia has been well documented [5], the rapid rise in Internet access through mobile devices has resulted in widespread growth in emerging market economies [5,6]. Although members of the general public were the early adopters of using the Internet and social media for their health care needs, health professional students have expressed a similar interest [7-10]. Globally, over 90% of university students actively use social media informally to create and share learning resources and to seek and provide moral support to one another [3,7-12]. However, given that 82% of patients around the world are interested in using Web-based mediums for health care purposes in the future [6], it highlights the importance of health care professional trainees learning to use these platforms to communicate professionally, academically, and clinically. So, how should academic institutions then prepare their students to have the skills and experience necessary to use social media to engage the public in the future?

A recent study demonstrated that medical students engaged in a course about social media and e-professionalism aided in the awareness of positive and negative uses of social media in a professional and educational environment [13]. Additionally, the majority of students made immediate changes to their social media use and reported that it would impact future Web-based behavior [13]. The use of social media in health care education has mainly been an area of increasing interest as a means of better engaging and enhancing the learning of students through methods outside the traditional didactic methodology, which relies on instructive teaching and passive learning. A number of studies have been conducted to investigate the ways in which the health care students informally use social media for educational purposes [14]. The results identify efficient communication with educators, peer collaboration, and small-group learning and sharing resources as key strengths [3,10]. Learners also use social media platforms to supplement their learning outside class, revisit key concepts, and view examples of physical exam skills [15,16]. Meanwhile, some health educators are starting to use social media formally as a method of delivering curricula and building student workplace competencies, including virtual journal clubs, reflective blogging, and microblogging platforms to enhance clinical decision making in a critical-care and team-learning setting [17-20]. Although the body of evidence investigating the effectiveness of using social media formally as a teaching tool is small, results show that social media use tends to lead to greater engagement, more active participation, and increased opportunities for feedback [7,12,14,15,17,18,21,22].

Adapting to new technologies and demands on time were identified as challenges to social media integration into education by educators and students [2,14,23-25]. Despite some integration of social media as an educational tool, broad-based adoption among educators has been slow. Apparent additional risks and challenges such as introducing a distraction during lectures or tutorials, difficulties with maintaining professionalism and patient confidentiality, legal implications of sharing information, and student exposure to low-quality health care information have been postulated as the reasons for the lag in adoption [2,7]. However, there have been a few studies that quantify these issues on a global scale. Hence, the purpose of this survey was to compare and contrast attitudes toward the use of social media as an educational tool with faculty who do and do not currently use social media in their teaching practice to determine the levels of awareness of social media policies and guidelines and to discern whether the various barriers articulated in the literature actually apply in practice for these international faculty members.

## Methods

### Study Design and Instrument Development

We conducted a global cross-sectional Web-based survey of 8 member institutions (see Table 1) from the Universitas 21 (U21) Health Sciences Consortium. U21 Health Sciences is a group of universities collaborating to explore health science education, research opportunities, and social transformation. The 8 participating institutions self-selected to take part in the “Social Media for Education in Health” project.

The research team included a faculty and student representative from each participating university, and they jointly developed a 24-question survey. Content for the first draft of the survey instrument was derived from the existing literature [26-30] and discussions among the research team. Furthermore, the draft was sent to global representatives from diverse health care disciplines, and feedback from experts in the health informatics/communications field and a statistician with experience in survey design was gathered before finalizing the survey. The final survey was constructed using FluidSurveys (Survey Monkey), a Freedom of Information and Protection of Privacy Act-compliant software, and ethical approval was obtained from research ethics boards of all participating institutions.

The site-specific faculty representative disseminated the survey to the members of their university community through electronic mailing lists targeting faculty, staff, and students. Although this survey was distributed to students and faculty, given the purpose of this study, only faculty responses were used. Response to the survey was accepted as informed consent, and responses were anonymous. Respondents were allowed to select more than one choice for nondemographic questions, where applicable. The survey was administered from July to December 2014. Inclusion criteria for the study required respondents to have identified as educators and reported their frequency of social media use. Respondents who only filled out demographic data were excluded.

### Data and Statistical Analysis

Data were downloaded from the Fluid Surveys platform into a Microsoft Excel spreadsheet and transferred to Stata/SE version 13 for analysis. Survey questions with an option for open text responses were coded into existing categories where applicable, or a new category was created as needed. Descriptive statistics were conducted on demographic data with continuous variables (age) expressed as a mean and standard deviation and categorical variables (gender and university affiliation) expressed as frequencies and percentages. Differences in the distributions of demographic variables were examined using a chi-square test (or Fisher exact test) for categorical variables or a *t*-test for age.

The chi-square test (or Fisher exact test where appropriate) was also applied and a *P* value of <.05 was considered to be statistically significant to examine the relationship between the frequency of social media use and barriers to the use of social media for health education, factors influencing decisions to use social media in teaching practice, capacity of social media to improve interactions among students/educators, and the type of social media currently used. The distribution of responses was similar on most questions for those who selected never and rarely as their frequency of social media use for educational purposes and similarly, for those who selected sometimes, often, and almost always. As such, the data were collapsed into two groups for all analyses, which were “nonuser” (never/rarely) and “user” (sometimes/often/almost always) for ease of interpretation.

## Results

Health educators from 8 global institutions and a variety of health disciplines, including nursing, public health, medicine, pharmacy, dentistry, and physiotherapy, responded to the survey. The survey response rate was reported individually by each university and ranged from 4% to 46%, with data from some institutions missing. Respondents were divided into two groups, users and nonusers, based on the frequency of social media use in educational practice. Of the 270 respondents, 143 (52.9%) were users, and 127 (47.0%) were nonusers. There was a statistically significant difference in the mean age of users compared with nonusers (43.8 vs 46.3 years; *P*=.045; Table 1).

### Perceived Barriers and Influencing Factors

Table 2 shows that among nonusers, the greatest perceived barriers to the use of social media in health professional education were a lack of understanding of how to integrate social media in their teaching practice (91/127, 71.7%), lack of departmental support (69/127, 54.3%), uncertainty on department policies (71/127, 55.9%), and lack of technical skills to use social media (71/127, 55.9%). Additionally, 41 out of 127 nonusers (32.3%) did not see the value of using social media in health education, which considerably differed in the proportion of users (7/143, 4.9%; *P* ≤.001). The two groups significantly differed on their attitudes to all barriers, except on concerns about professionalism (73/127, 57.5% vs 70/143, 49.0%; *P*=.18).

**Table 1:** Demographics.

Demographics		Users	Nonusers	Total	*P* value
**Age, mean (SD)**		**N=138**^a^	**N=121**^a^	**N=259**^a^	.045
	Age in years	43.8 (9.3)	46.3 (10.3)	45.0 (9.8)	
**Gender, n (%)**		**N=141**^a^	**N=125**^a^	**N=266**^a^	.07
	Male	44 (31.2)	53 (42.4)	97 (36.5)	
	Female	97 (68.8)	72 (57.6)	169 (63.5)	
**University affiliation, n (%)**		**N=142**^a^	**N=126**^a^	**N=268**^a^	<.001
	Fudan University, Shanghai, China	27 (67.5)	13 (32.5)	40 (14.9)	
	Instituto Tecnológico de Monterrey, Nuevo Leon, Mexico	17 (85.0)	3 (15.0)	20 (7.5)	
	University of Birmingham, West Midlands, United Kingdom	9 (26.5)	25 (73.5)	34 (12.7)	
	University of British Columbia, Vancouver, Canada	23 (62.2)	14 (37.9)	37 (13.8)	
	University College of Dublin, Dublin, Ireland	5 (71.4)	2 (28.6)	7 (2.6)	
	University of Hong Kong, Pokfulam, Hong Kong	18 (75.0)	6 (25.0)	24 (9.0)	
	University of Melbourne, Victoria, Australia	8 (38.1)	13 (61.9)	21 (7.8)	
	University of Nottingham, Nottingham, United Kingdom	35 (41.2)	50 (58.8)	85 (31.7)	

^a^Note: Denominator varies slightly because of missing data.

^b^SD: standard deviation.

**Table 2:** Barriers to the use of social media for health professionals’ education (in descending order of “Nonuser” group responses).

Factors	Users (N=143) n (%)	Nonusers (N=127) n (%)	*P* value
Do not understand how to incorporate social media into teaching/ learning	35 (24.5)	91 (71.7)	<.001
Concerns about professionalism	70 (49.0)	73 (57.5)	.18
Unsure about department’s policies related to the use of social media	54 (37.8)	71 (55.9)	.005
Lack the technical skills to use social media tools	42 (29.4)	71 (55.9)	<.001
Department does not offer support for the use of social media in health education	59 (41.3)	69 (54.3)	.01
Do not see the value of using social media in health education	7 (4.9)	41 (32.3)	<.001
Department prohibits or actively discourages the use of social media in health education	5 (3.5)	14 (11.0)	.02

**Table 3:** Factors influencing decisions to use social media in teaching/learning practice (in descending order of “Nonuser” group responses).

Influencing factor	Users (N=143) n (%)	Nonusers (N=127) n (%)	*P* value
Evidence that learning is enhanced through the use of social media tools	96 (67.1)	73 (57.5)	.13
Ability and knowledge in the use of social media tools	78 (54.5)	65 (51.2)	.63
Support from experts in the use of social media to design teaching strategies/modules	52 (36.4)	62 (48.8)	.048
Fit of social media tools to the style of teaching/learning	65 (45.5)	61 (48.0)	.72
Improved learner satisfaction with the course	97 (67.8)	50 (39.4)	<.001
Peers using social media technologies in their classrooms	65 (45.5)	47 (37.0)	.18
Improved student evaluations of the course	56 (39.2)	44 (34.6)	.45
Course/Department coordinator suggesting the use of social media technologies in the classroom	45 (31.5)	40 (31.5)	>.99
Compatibility of social media technologies with the devices in use within classroom	65 (45.5)	33 (26.0)	.001

Table 3 describes the factors most likely to influence a nonuser to use social media in their teaching practice, which include (1) evidence demonstrating that learning is enhanced through the use of social media (73/127, 57.5%) and (2) their own ability and knowledge in using the associated technology (65/127, 51.2%). Additionally, support from the experts in the field of using social media for educational purposes to help design teaching strategies was a factor that was significantly more important for nonusers (62/127, 48.8% vs 52/143, 36.4%; *P*=.048).

Meanwhile, users have also rated supportive evidence to illustrate the enhanced learning from the use of social media as an important influencing factor (96/143, 67.1%). However, in stark contrast to nonusers, they were significantly more likely to be influenced by improved learner satisfaction (97/143, 67.8% vs 50 out of 127, 39.4%; *P* ≤.001).

Finally, users agreed that social media has the capacity to positively impact educational practices, whereas nonusers were significantly more skeptical of its ability to improve student learning (116/136, 85.3% vs 70/125, 56.0%; *P* ≤.001) and increase faculty-to-student interactions (127/140, 90.8% vs 81/126, 64.3%; *P* ≤.001). However, nonusers tended to agree with the users on social media’s use in health education in increasing interactions among the student population, although there was still a significant difference (122/137, 89.1% vs 96/125, 76.8%; *P*=.02)—Data not shown in the table.

### Knowledge of Policies and Guidelines

Both users and nonusers reported not being trained on social media–related policies and guidelines, with only 11.5% (16/139) and 5.8% (7/120), respectively, having been provided prior training (*P*=.18). Educators from both groups who did receive training reported having increased confidence in using social media for educational purposes (14/16 users, 87.5% and 5/7 nonusers, 71.4%; *P*=.56). Of those educators who had not been trained, 85.5% of users (106/124) and 73.5% of nonusers (83/113) would like to be provided with training on social media use; however, users were significantly more likely to want training compared with nonusers (*P*=.03).

## Discussion

### Principal Findings

Our survey found that almost three-quarters of educators only used social media as an educational tool “sometimes” or less often. Students’ use of social media for health education is overwhelmingly higher, with almost the same proportion using social media often or always [31-35]. There is a clear discrepancy as students’ usage of social media to enhance their education informally is growing disproportionally faster [31,32]. Creating categories of users and nonusers provided a means of comparing the attitudes of educators and understanding the factors that contribute to the differences in adoption.

Our findings suggest that the differences in mean age of the user and nonuser groups are statistically significant. In practicality, the 2.6-year difference in mean age and range of ages in each group may not be contextually relevant. Hence, unlike previous studies that suggested age and gender as major factors for the lack of broad-based adoption, our sample does not demonstrate strong demographic differences between users and nonusers [36,37]. However, the data from our study does suggest that the two groups have unique perceived challenges and needs and engaging them to adapt social media into their educational approaches will require very different approaches, which are previously unreported in the literature.

Nonusers perceived their greatest barrier to be a lack of comfort and technical skills. Therefore, evidence-based recommendations on principles, best practices, and successful strategies can be helpful to nonusers who are not confident in educational social media usage [31,36,38]. Although the rapid evolution of social media platforms could make the technological aspect more approachable, which would improve nonuser adoption, the growing number of competing tools could make the process of choosing a platform daunting and overwhelming [39,40]. Hence, greater foundational support from experienced peers, information technology departments, and industry experts on the basics of integrating social media tools in the delivery of content may improve uptake among nonusers. However, some nonusers may still not see the value of social media; consequently, institutions may want to recognize differences in opinions and encourage open debate and discussion among their faculty about the strengths and weaknesses of social media usage.

By comparison, users strongly believe in the capacity of social media to improve student learning and faculty and peer interactions with students, highlighting the importance of providing them with new evidence-based ways to increase engagement and supporting their efforts to incorporate innovative methods into their educational practice [41]. Unsurprisingly, users were more influenced to increase social media use in the academic setting by student-centric factors such as improved learner satisfaction and student evaluation, suggesting that feedback and active participation from students when educators do integrate social media into their content delivery could encourage more frequent use, and potentially more innovative or adventurous usages.

The users and nonusers did share commonalities; both were greatly influenced by evidence that learning is enhanced through social media integration and resources to aid educators increase their abilities and knowledge of social media–based teaching tools. As the body of evidence is continuously growing, the need for further high-quality literature is underscored by the need for effective dissemination of results [14,42]. Additionally, given that less than 11% of educators from both groups have received training on the policies and guidelines of social media use in the academic setting, institutions may focus on making their policies and guidelines clear and accessible through training and open forums for discussion at faculty development sessions [36,43].

We also found that both groups shared similar concerns on the impact that integrating social media in health education would have on professionalism. In the new media age, the distinction between personal and professional Web-based content is blurry, and the definition of appropriate behavior remains uncertain. Within the health care context, patients are likely to judge health professionals on their Web-based persona, which may in turn affect trust and adherence to advice [44]. Simultaneously, societal uptake of social media and general patient interest in connecting and engaging with health care professionals over social media is growing rapidly [45,46]. Thus, concern over the professional identity of a health care professional is a complex issue. However, engaging with these tools early, and in the “safer” educational context, will give educators and students the opportunity to experiment, experience, and reflect on how best to meet their professions’ standard and public expectations [43,47].

Compared with the existing literature that largely comprises postulated barriers, our study substantiated some but not others. Although, professionalism, legal implications, and time investment are all important issues, they are of secondary importance to technological support, learner engagement, and clarity of institutional policies and guidelines. Hence, our study demonstrates an unreported set of issues to consider and the practical nature of educators’ priorities when approaching social media in health education.

### Limitations

This study has several limitations. As with most survey-based studies, our results may be subject to construct bias. However, an extensive review process was carried out a priori to minimize risk. Additionally, because faculty representatives at each institution disseminated the survey, there may have been inconsistencies leading to difficulties in determining the response rate and introducing potential bias.

The sample size was small and derived from voluntary participation; hence, it may have been limited by faculty population size, institutional stance on social media use, and strength of interest or opinion, thereby leading to potential type 2 error or insufficient power. Finally, the institutions self-selected to be participants; hence, our results are likely not generalizable to all health professional programs.

### Conclusions

In conclusion, our survey results have demonstrated that adoption of social media as a teaching tool is not uniform for all faculty members but necessitates targeted strategies for current users and nonusers. The two groups have unique attitudes, needs, and motivations that need to be addressed. Furthermore, both groups need clear evidence that demonstrate effectiveness of social media as an educational strategy and thorough understanding of the institutional boundaries of social media use. Therefore, institutions need to discern the mix of users and nonusers that exists in their faculty population before instituting change management strategies to engage them in social media use in health professional education.

With health education moving away from the conventional approach of didactic knowledge transmission, social media could be an effective modality to employ a Socratic methodology where students and educators jointly collaborate to facilitate enhanced learning. Our findings suggest that the majority of users and nonusers are open-minded to incorporating social media into their teaching practice, and so they should be encouraged to do so, in accordance to their respective needs.

### Ethical Approval

The ethics application was submitted to the University of British Columbia, and each of the other 7 participating institutions (Fudan University, China; Instituto Tecnológico de Monterrey, Mexico; University of Birmingham, the United Kingdom; University College of Dublin, Ireland; University of Hong Kong, Hong Kong; University of Melbourne, Australia; and University of Nottingham, the United Kingdom) used the approval to receive institutional departmental authorization before the administration of the survey.

## Abbreviations

SD: standard deviation
